# Cognitive penetration and the gallery of indiscernibles

**DOI:** 10.3389/fpsyg.2014.01527

**Published:** 2015-01-08

**Authors:** Bence Nanay

**Affiliations:** ^1^Centre for Philosophical Psychology, University of AntwerpBelgium; ^2^Peterhouse, University of CambridgeCambridge, UK

**Keywords:** perception, cognitive penetration, aesthetic value, indiscernibles, attention

Here is Danto's gallery of indiscernibles thought experiment (Danto, 1981, p.1)—a thought experiment that radically transformed the kind of questions aesthetics and the philosophy of art asks today. Imagine a gallery of indiscernible canvases that are all monochrome red of the same shade and of the same size. While the observable properties of all these artworks are the same, their “meaning” and aesthetic value can be very different: if one of the paintings, made by a counterrevolutionary Russian émigré is called “Red Square” and the other one is called “The Israelites crossing the Red Sea,” then these two paintings, in spite of being indistinguishable, will have very different aesthetic value. Thus, aesthetic value is only loosely (if at all) related to perception.

Let us examine this argument more closely. First, I need to introduce a bit of terminology: I call a property “aesthetically relevant” if attending to this property changes one's aesthetic evaluation (Nanay, 2015). Aesthetically relevant properties are abundant. If I attend to the arrangement of the small red patches in a Corot landscape, it can change my experience and assessment of the balance of the picture. And if I attend to the second violin in a string quartet, it can also change my experience of the entire movement. Danto's argument is supposed to establish that there is only a loose connection between aesthetically relevant properties and perception.

Danto oscillates between two arguments in his exposition of the gallery of indiscernibles, one weaker and unproblematic (and somewhat trivial), the other stronger and problematic. Here is the weaker one (I take P1 to be the painting by the counterrevolutionary Russian émigré, “Red Square” and P2 to be the painting called “The Israelites crossing the Red Sea”):

(1^*^) The observable properties of P1 are the same as the observable properties of P2.
(2^*^) The aesthetically relevant properties of P1 are different from the aesthetically relevant properties of P2.
  ---------------------------------------
(3^*^) Aesthetically relevant properties do not supervene on observable properties.

I take it that no-one would want to deny (1^*^), (2^*^) or (3^*^). Nor should any of these claims strike anyone as particularly surprising or novel. Everyone but really strict formalists would accept that at least some aesthetically relevant properties do not supervene on observable properties. Danto must have meant something stronger. In fact, he did mean something stronger, namely, the following:

1. The perceptual experience of P1 is the same as the perceptual experience of P2.
2. The aesthetically relevant properties we attribute to P1 are different from the ones we attribute to P2.
  ---------------------------------------
3. The attribution of aesthetically relevant properties does not supervene on our perceptual experience.

Note the difference between this argument for (3) and the one for (3^*^) above. (3^*^) is about the logical relation between aesthetically relevant properties and observable properties, whereas (3) is about the logical relation between two kinds of mental states (the attribution of aesthetically relevant properties and perceptual experiences). Very different claims (and very different arguments) indeed.

We have good reasons to hold (2). And (3) clearly follows from (1) and (2). The problem with Danto's argument for (3) is premise (1). I will argue that premise (1) is false. But, again, (1^*^) is true:

(1^*^) The observable properties of P1 are the same as the observable properties of P2.

The observable properties of the two paintings are, by supposition, exactly the same. In fact, Danto goes further and says that even all physical properties of the two pictures are identical. But (1^*^) does not imply (1). Two objects may have the very same observable properties, nonetheless, one's perceptual experience of them may be very different. (1^*^) only implies (1) if we add a further premise, one that Danto took for granted (see esp. Danto, 2001a,b; see also Fodor, 1993):

(1^**^) Perceptual experiences are not cognitively penetrable.

Danto's argument only goes through if we add this extra premise (see also Wollheim, 1993; Margolis, 1998, 2000; Lamarque, 2010; Nanay, 2015). If we block (1^**^), we have no reason to hold (1) and then we have no reason to hold (3). Here is the reason why blocking (1^**^) jeopardizes the whole argument. If perceptual experiences are cognitively penetrable (i.e., if (1^**^) is false), then the difference in the title of the pictures can and will bring about a perceptual difference. As a result of my non-perceptual state of reading the title, my perceptual experience will be different. But then (1) is false, which means that there is no reason to hold (3).

And, as it turns out, there is very strong empirical evidence against (1^**^)—both as a general claim and as a claim in this specific context (Goldstone, 1995; Hansen et al., 2006; Lupyan and Spivey, 2008; Lupyan et al., 2010; Lupyan and Ward, 2013; Nanay, 2013a,b, but see also Firestone and Scholl, 2014 for a critical analysis). There are top-down processes that influence perceptual processing as early as the primary visual cortex (Gandhi et al., 1999) or the thalamus (O'Connor et al., 2002). Here is an old experiment, very much known at the time when Danto gave this argument (Delk and Fillenbaum, 1965): if we have to match the color of a picture of an orange heart to color samples, we match it differently (closer to the red end of the spectrum) from the way we match the color of a picture of some other, orange shapes. This shows that our recognition of the object in question (the heart) influences the color we experience it as having. In general, one's experience is not determined in a bottom-up manner by the perceptual stimulus: it depends on language, attention, the contrast classes and one's expectations (see Hansen et al., 2006; Lupyan and Ward, 2013).

The defender of Danto's claim would need to deny that we have any reasons to question (1^**^). The concept of “cognitive penetrability” has been severely debated in the last decades and depending on how one defines this concept (see Siegel, 2011; Macpherson, 2012 for summaries), it may not be too farfetched to retain *some* sense in which perceptual experiences are not cognitively penetrable—in which case, we can salvage (1^**^) and with it Danto's argument. While it may indeed be true that there may be some sense in which perceptual experiences are cognitively impenetrable (Pylyshyn, 1999), the sense of cognitive impenetrability that would be required for Danto's argument to go through is not one of these.

Here is why (Levin and Banaji, 2006): Two pictures of identical (mixed race) faces were shown to subjects—the only difference between them was that under one the subjects read the word “white” and under the other they read “black.” When they had to match the color of the face, subjects chose a significantly darker color for the face with the label “black”^1^.

This experiment has the exact same structure as Danto's thought experiment: the two visual stimuli share all their observable properties—just like the two canvases. But, crucially, our experience of the two stimuli are different—we see one as being darker than the other. Similarly, when we see the painting called Red Square, our perceptual experience of the painting may be colored by our previous exposure of the soviet red flag, for example—something that is missing from our perceptual experience of the other painting. While (1^*^) is true, (1) is false. But if (1) is false, then we have no reason to hold (3).

The gallery of indiscernibles thought experiment is based on an empirically inadequate way of thinking about perception. On any empirically adequate ways of thinking about perception, we have no reason to take the gallery of indiscernibles seriously.

The structure of my argument was that we can bypass the thorny question of what counts as cognitive penetration because whatever sense it is in which our perception of the faces in the Levin and Banaji experiment is penetrable, it is exactly the same sense in which our perception of the artworks in Danto's thought experiment is penetrable. But it is important to highlight that this is a very weak sense of cognitive penetrability—so much so that it wouldn't even count as cognitive penetrability under many formulation of cognitive penetrability (e.g., Siegel, 2011, p. 204) because all it implies is that our perceptual experience is subject to top-down attentional influences—something even those who deny the cognitive penetrability of perception would accept (see Pylyshyn, 1999).

Taking the painting to be about Russia or about the Red Sea influences what properties of the picture we are attending to. But as the inattentional blindness findings show, what properties we are attending to very much influences our perceptual phenomenology (and we also know that attention can modulate even the earliest stages of visual processing, the primary visual cortex, see Gandhi et al., 1999). But then our perceptual experience of the two paintings in the Gallery of Indiscernibles thought experiment is very different because we are attending to them very differently.

Crucially, the difference in attention brings about a difference in *perceptual* phenomenology. To see this, it may be helpful to consider the following example (e.g., Nickel, 2007; Nanay, 2010): You are looking at a 3 × 3 grid of squares against a white background. First experience: you are attending to the corner and the center squares. Second experience: you are attending to the remaining four squares. The two experiences are phenomenally different—different squares seem prominent.

In other words, different ways of attending to very simple figures of this kind changes our perceptual phenomenology. But then presumably different ways of attending to Danto's indistinguishable canvases would also make our perceptual phenomenology of these experiences different. It is important to emphasize that this phenomenal difference is a difference in perceptual phenomenology. There are some more controversial cases, like the duck-rabbit illusion, where it is also true that attention to the rabbit vs. attention to the duck very much influences our phenomenology. But in the duck-rabbit case one could object that what changes is not something perceptual: that it is the interpretation of the scene that changes (Brewer, 2007, p. 93).

In the case of the 3 × 3 grid, however, the phenomenal differences (e.g., salience) are clearly properties that are perceptually experienced (even by the thinnest accounts of perceptual experience). And we can run the same explanatory scheme for why our perceptual experience of P1 is different from our perceptual experience of P2—in one, but not in the other, our experience of the red of the canvas is colored by the association of the red of the Soviet flag, for example. But then the difference in the attribution of aesthetically relevant properties is accompanied by a difference in our perceptual experience. Danto's argument from the Gallery of Indiscernibles fails.

We can now conclude that the attribution of aesthetically relevant properties, while it does not have to be a perceptual attribution, very much supervenes on one's perceptual experience: if there is a difference in the attribution of aesthetically relevant properties, there must also be a difference in one's perceptual experience. This restores the nice and tight connection between aesthetically relevant properties and perception: while not all aesthetically relevant properties are perceived, they all have very serious perceptual consequences.

## Conflict of interest statement

The author declares that the research was conducted in the absence of any commercial or financial relationships that could be construed as a potential conflict of interest.

## Footnotes

^1^There has been some controversy about the Levin and Banaji (2006) findings, especially about their first experiment (e.g., Firestone and Scholl, 2014). But the experiment I want to use here is not their first but their second experiment (where two faces are identical in all respects except for the label under them). While there are some methodological issues about this experiment as well (about whether the label influences our experience or merely the matching task performed, see Lupyan, in press, footnote 4), I want to bracket these for the purposes of this discussion. If the reader is not fully convinced by this experiment, she can use some of the other, less debated empirical findings, (see Goldstone, 1995; Hansen et al., 2006; Lupyan and Spivey, 2008; Lupyan et al., 2010; Lupyan and Ward, 2013).
